# Quantitating Age‐Related BMD Textural Variation from DXA Region‐Free‐Analysis: A Study of Hip Fracture Prediction in Three Cohorts

**DOI:** 10.1002/jbmr.4638

**Published:** 2022-07-15

**Authors:** Mohsen Farzi, Jose M. Pozo, Eugene McCloskey, Richard Eastell, Nicholas C. Harvey, Alejandro F. Frangi, Jeremy Mark Wilkinson

**Affiliations:** ^1^ Department of Oncology and Metabolism The University of Sheffield Sheffield UK; ^2^ The Medical Research Council (MRC)‐Arthritis Research UK Centre for Integrated Research into Musculoskeletal Ageing (CIMA), The University of Sheffield Sheffield UK; ^3^ Centre for Computational Imaging and Simulation Technologies in Biomedicine (CISTIB) The University of Leeds Leeds UK; ^4^ The MRC Lifecourse Epidemiology Centre University of Southampton Southampton UK; ^5^ NIHR Southampton Biomedical Research Centre University of Southampton and University Hospital Southampton UK

**Keywords:** AGING, DXA, BMD, HIP FRACTURES, OSTEOPOROSIS

## Abstract

The risk of osteoporotic fracture is inversely related to bone mineral density (BMD), but how spatial BMD pattern influences fracture risk remains incompletely understood. This study used a pixel‐level spatiotemporal atlas of proximal femoral BMD in 13,338 white European women (age 20–97 years) to quantitate age‐related texture variation in BMD maps and generate a “reference” map of bone aging. We introduce a new index, called Densitometric Bone Age (DBA), as the age at which an individual site‐specific BMD map (the proximal femur is studied here) best matches the median aging trajectory at that site in terms of the root mean squared error (RMSE). The ability of DBA to predict incident hip fracture and hip fracture pattern over 5 years following baseline BMD was compared against conventional region‐based BMD analysis in a subset of 11,899 women (age 45–97 years), for which follow‐up fracture records exist. There were 208 subsequent incident hip fractures in the study populations (138 femoral necks [FNs], 52 trochanteric [TR], 18 sites unspecified). DBA had modestly better performance compared to the conventional FN‐BMD, TR‐BMD, and total hip (TOT)‐BMD in identifying hip fractures measured as the area under the curve (AUC) using receiver operating characteristics (ROC) curve analysis by 2% (95% confidence interval [CI], −0.5% to 3.5%), 3% (95% CI, 1.0% to 4.0%), and 1% (95% CI, 0.4% to 1.6%), respectively. Compared to FN‐BMD *T*‐score, DBA improved the ROC‐AUC for predicting TR fractures by ~5% (95% CI, 1.1% to 9.8%) with similar performance in identifying FN fractures. Compared to TR‐BMD *T*‐score, DBA improved the ROC‐AUC for the prediction of FN fractures by ~3% (95% CI, 1.1% to 4.9%), with similar performance in identifying TR fractures. Our findings suggest that DBA may provide a spatially sensitive measure of proximal femoral fragility that is not captured by FN‐BMD or TR‐BMD alone. © 2022 The Authors. *Journal of Bone and Mineral Research* published by Wiley Periodicals LLC on behalf of American Society for Bone and Mineral Research (ASBMR).

## Introduction

The assessment of bone quality by measuring bone mineral density (BMD) using dual‐energy X‐ray absorptiometry (DXA) is a cornerstone of osteoporosis management.^(^ ^1^, ^2^ ^)^ The inverse relationship between areal BMD (aBMD) and incident fracture,^(^ ^3^ ^)^ combined with clinical risk factors,^(^ ^4^ ^)^ is used to guide clinical management.^(^ ^5^, ^6^, ^7^, ^8^ ^)^ Data from several studies have shown that site‐specific measurement of BMD provides the best prediction of fracture risk at that site.^(^ ^9^, ^10^ ^)^ At the hip, a large meta‐analysis of several prospective studies showed a relative risk for hip fracture of 2.6 (95% confidence interval [CI], 2.0 to 3.5) per standard deviation (SD) of decrease in femoral neck (FN)‐BMD.^(^ ^3^ ^)^ However, data from the Study of Osteoporotic Fractures (SOF) also shows that almost half of all fragility hip fractures occur in individuals with a FN *T*‐score of > −1.5.^(^ ^10^, ^11^ ^)^ Further, FN‐BMD does not capture all determinants of bone strength in the proximal femur. For example, trochanteric (TR)‐BMD is associated with intertrochanteric femoral fractures independent of FN‐BMD,^(^ ^12^ ^)^ consistent with the observation that pixel‐summation and quantitation of BMD within one region of interest (ROI) give rise to better fracture prediction within that ROI versus BMD at another site,^(^ ^13^, ^14^ ^)^ even if the sites are contiguous. A further limitation of conventional BMD assessment is that the output metrics (*T*‐score and *Z*‐score) are not intuitive for patients, making the explanation of the results difficult for them to interpret and contextualize.

Alternative analytical approaches have been explored to address the low sensitivity of DXA in population‐attributable fracture risk prediction. At the lumbar spine, trabecular bone score (TBS)^(^ ^15^, ^16^ ^)^ is an analytic method that measures the rate of local variations in gray‐level from the two‐dimensional (2D) lumbar spine DXA image to provide an indirect index of three‐dimensional (3D) trabecular microarchitecture. Several studies, reviewed in a European consensus report by Harvey and colleagues,^(^ ^17^ ^)^ have demonstrated that TBS is a predictor of vertebral fracture independent of aBMD and provides complementary information on vertebral bone quality in diseases associated with fragility fractures. However, at the FN, the site of greatest disease burden in osteoporosis, studies have not yet identified an analytic approach that extracts greater clinically‐useful information from conventional DXA images than aBMD. Hip structural analysis (HSA) uses the distribution of mineral mass in a line of pixels across the bone axis to measure geometric properties of cross‐sections of bone (compiled to ~5 mm thickness) at that region.^(^ ^18^ ^)^ This approach provides estimates of bending strength, compressive strength, and buckling strength at discrete regions of interest in the plane of the DXA image. Lower bone strength measured with HSA was significantly linked with a higher tendency to fracture but its clinical utility in adequately predicting FN fractures beyond routine aBMD requires further study.^(^ ^14^, ^19^, ^20^, ^21^ ^)^ Yang and colleagues^(^ ^22^ ^)^ used DXA‐based structural engineering models to calculate lateral stress upon the hip with a sideways fall that provided better FN fracture prediction than aBMD but did not better predict TR fractures. Several machine‐learning‐based methods have also been proposed to quantitate texture features from pixel‐level DXA scans to improve hip fracture prediction.^(^ ^23^, ^24^, ^25^ ^)^ Despite their potential merits, the small number of fracture cases (*n* ≤ 50) is a significant limitation in these studies.^(^ ^23^, ^24^, ^25^ ^)^ Given the relatively low rate of hip fractures in the population (~2% to 5%), employing a discriminative learning approach is prone to overfitting errors.

We have previously reported a technique termed DXA Region‐Free Analysis (DXA‐RFA) to extract pixel‐level BMD from DXA datasets that describes bone loss occurring around hip joint prostheses.^(^ ^26^, ^27^, ^28^, ^29^ ^)^ We have recently used this method to develop a calibrated and validated spatiotemporal aging atlas of the native proximal femur.^(^ ^30^ ^)^ In the present study, we aimed to (i) introduce a more intuitive index, coined “Densitometric Bone Age” (DBA), derived from quantification of textural BMD variation using the developed aging atlas for the proximal femur; (ii) determine whether DBA can better predict incident hip fractures versus conventional FN‐BMD, TR‐BMD, and total hip (TOT)‐BMD *T*‐score; and (iii) determine whether DBA can predict the anatomic pattern of incident hip fracture (FN versus TR).

## Patients and Methods

### Study populations

The pseudoanonymized patient demographic, incident hip fracture, and imaging data described in this study were accessed from UK Biobank (approval 17881; July 09, 2018), the OPUS study,^(^ ^31^ ^)^ and the Medical Research Council (MRC)‐Hip study^(^ ^32^ ^)^ (Table 1). Ethics approval for these cohorts was obtained under institutional and national requirements, and all subjects provided written informed consent before participation. The participants comprised white women of European descent (MRC‐Hip study *n* = 5018, aged 75–97 years, mean ± SD = 80 ± 3.9 years; OPUS *n* = 213, aged 20–39 years, mean ± SD = 32 ± 5.3 years; and *n* = 1189, aged 55–79 years, mean ± SD = 67 ± 7.1 years; UK Biobank *n* = 6918, age 45–80 years, mean ± SD = 62 ± 7.3 years). All scans (*n* = 13,338) were used to develop the spatiotemporal BMD aging atlas, as detailed in our previous work,^(^ ^30^ ^)^ and to assess the relationship between DBA and FN‐BMD *T*‐score and *Z*‐score. The precision of DBA as a quantitative tool was estimated by analyzing 25 pairs of DXA scans in a subset of the OPUS cohort. The scan pairs were collected on the same day with patient repositioning between acquisitions.

**Table 1:** Characteristics of the Patient Populations Participating in This Study

Population	*n*	Gender	Age span (years)	Age (years) mean ± SD	Body mass index mean ± SD (kg/m^2^)	Number of hip fractures
UK Biobank Study	6918	Female	45–80	62 ± 7.3	25.7 ± 4.7	27
MRC‐Hip Study	5018	Female	75–97	80 ± 3.9	26.6 ± 5.2	181
OPUS study‐group1	1189	Female	55–79	67 ± 7.1	26.2 ± 5.2	–
OPUS study‐group2	213	Female	20–39	32 ± 5.3	24.2 ± 5.0	–

The cohort used to evaluate the ability of DBA to predict incident hip fractures comprised (*n* = 5018) participants in the MRC‐Hip study followed for 5 years after baseline DXA measurement and (*n* = 6881) participants in the UK Biobank study followed for a mean of 4.4 years. A total of 181 participants in the MRC‐Hip study and 27 in the UK Biobank study suffered an incident hip fracture during the follow‐up period. In the MRC‐Hip study, hip fracture types were also assessed by plain radiography, with 123 cases reported at the FN, 40 patients at the TR region, and 18 instances without defined fracture pattern. In the UK Biobank (UKBB) study, hip fracture types were assessed using a combination of International Classification of Diseases and Related Health Problems, 10th Revision (ICD‐10) (S72.0, S72.1) hip fracture and OPCS Classification of Interventions and Procedures version 4 (OPCS4) procedure codes. Fifteen fractures were classified as in the FN, and 12 were TR.

In this study, scans were collected either on a Hologic QDR 4500A (Hologic Inc, Waltham, MA, USA) in the MRC‐Hip and OPUS studies or an iDXA Lunar GE scanner (GE Healthcare, Madison, WI, USA) in the UKBB study. To amalgamate data from two manufacturers, linear calibration parameters were estimated using the quantile matching regression technique, as previously validated.^(^ ^30^ ^)^ In brief, *n* = 406 white British women matched for age and body mass index (BMI) were selected for each scanner. Next, at each pixel coordinate, the linear calibration parameters, ie, the slope and the intercept, were estimated such that the BMD distribution in each group was matched between scanners.

### Statistical Methods

#### DBA estimation

**Fig. 1:**
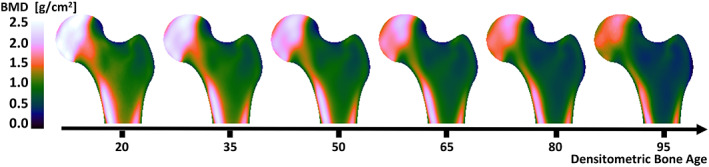
Median spatial BMD maps for a population of white European women (*n* = 13,338).^(^ ^30^ ^)^ Note that the bone aging trajectory is a continuum, and the six bone maps shown here at equal intervals of 15 years are for visual purposes.

**Fig. 2:**
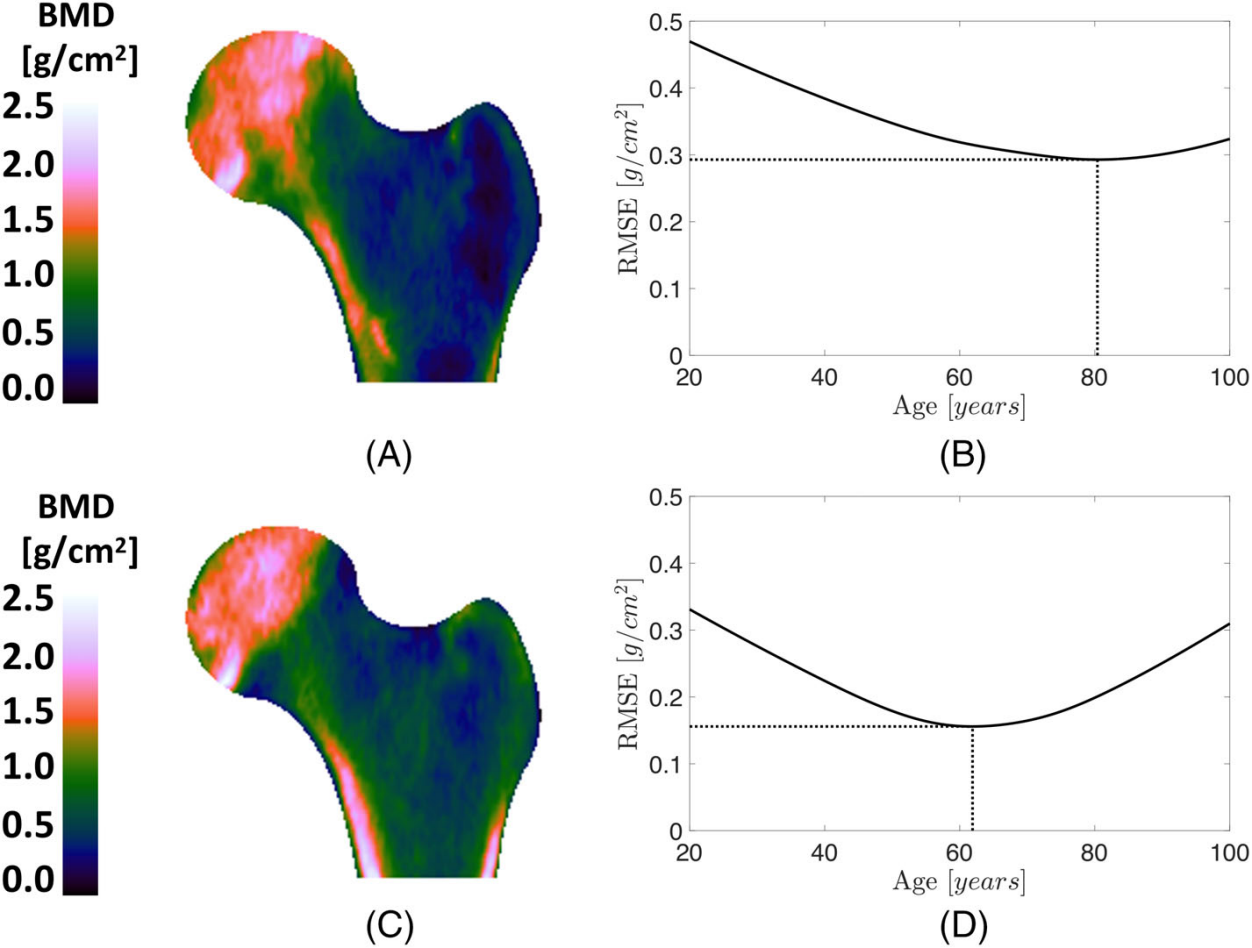
A visual example of spatial BMD maps to differentiate between fractured and control subjects with similar neck BMD values. (*A*) The bone map for a woman aged 75.8 years with FN‐BMD of 0.5860 g/cm^2^ who experienced a trochanteric fracture following the baseline measurement. (*C*) The bone map for a nonfracture subject with similar age (75.9 years) and FN‐BMD (0.5900 g/cm^2^). Despite similar age and FN‐BMD, the widespread trochanteric bone loss, which is not captured by FN‐BMD, resulted in a trochanteric fracture for the first subject. (*B,D*) The RMSE between the BMD maps in *A* and *C* and the median BMD maps in Fig. 1, respectively. DBA is the age at which the RMSE is minimum. The associated DBA was 80 and 62 years for the top and bottom subjects, respectively. DBA = densitometric bone age; RMSE = root mean squared error.

The spatiotemporal atlas of BMD in the proximal femur was developed as described previously.^(^ ^30^ ^)^ In brief, BMD maps were obtained for each DXA scan with an isotropic spatial resolution of 0.5 × 0.5 mm^2^ using either Hologic Apex v3.2 (Hologic, Inc.) or Lunar enCORE v16 (GE Healthcare) proprietary software, respectively. Automatic segmentation of the proximal femur was performed by selecting 65 landmark points around the bone contour using the “Bone‐Finder v.1.2.0” software developed by Lindner and colleagues.^(^ ^33^ ^)^ A standard template composed of ~16,000 pixels was generated by averaging over all segmented femurs. To remove the morphological variation between scans, all DXA scans were then warped into the template using a thin‐plate spline (TPS) registration method using in‐house Matlab software v9.7.0.1190202 R2019b (MathWorks, Cambridge, MA, USA).^(^ ^34^ ^)^ Age‐specific BMD distribution at each individual pixel was estimated smoothly using the R‐package VGAM (R Foundation for Statistical Computing, Vienna, Austria; https://www.r-project.org/).^(^ ^35^ ^)^ The resulting atlas allows probabilistic estimation of age‐specific pixel‐level BMD at any given anatomic site within the proximal femur across the studied age range. Here, we define the “normal bone aging” trajectory by estimating the median BMD map in the population (Fig. 1). The underlying assumption of the bone aging trajectory is that all subjects follow a consistent path across the chronological aging spectrum but at a different speed due to relatively accelerated/decelerated rates of bone loss during aging. With this definition, DBA is the age at which the root mean squared error (RMSE) between the median BMD map and the individual BMD map is smallest (or minimum) (Fig. 2A‐D). Note that DBA depends only on the spatial texture of BMD maps rather than the chronological age.

### Precision analysis

DBA is a quantitative measurement technique. A subset of the OPUS cohort (*n* = 25) was scanned twice on the same day, with patient repositioning between scans to assess its precision. For each scan, DBA was computed independently. The coefficient of variation (CV) was then calculated as the root mean square standard deviation divided by the mean of paired measurements.^(^ ^36^ ^)^ To visualize the agreement between measurements, Bland‐Altman plots were employed.

### DBA and its relationship with the FN‐BMD *T*‐score and *Z*‐score

**Fig. 3:**
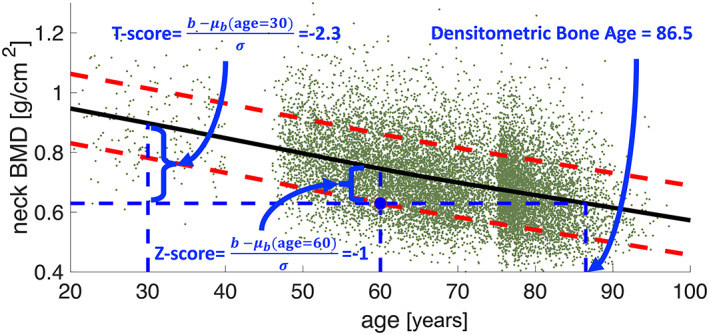
The analogy between the *T*‐score, *Z*‐score, and DBA. The solid black line shows the average FN‐BMD; the red dashed lines show 1 SD. The blue dot represents an individual aged 60 years with FN‐BMD = 0.63 g/cm^2^. DBA is the age at which the measured BMD equals the average BMD; ie, 86.5 years. The BMD distribution is normal for areal FN‐BMD because of the pixel averaging in the FN region, and the median and mean trajectories overlapped here. When DBA is computed based on pixel BMD values, the BMD distribution is no longer normal, and the median trajectory is used instead. DBA = densitometric bone age; SD = standard deviation.

DBA is similar in principle to the conventional *T*‐score and *Z*‐score. An individual's “score” is defined by comparison against a set of reference values from the population. Figure 3 demonstrates this analogy for aBMD at the FN. Note that similar to FN‐BMD *T*‐score and *Z*‐score, DBA is also a linear function of FN‐BMD. Figure 3 shows the bone aging distribution and trajectory for FN‐BMD, with each point representing an individual subject from the population. Similarly, for *n* = 16,000 pixels representing the whole proximal femur, the bone aging trajectory would be a smooth nonlinear curve in this high‐dimensional space. When DBA is computed over approximately *n* = 16,000 pixels on the template, the spatial BMD texture also contributes to calculating bone age. The Pearson coefficient correlation *r* is reported to assess the relationship between DBA and FN‐BMD *T*‐score and *Z*‐score. All statistical tests were performed in MATLAB v9.7.0.1190202 (R2019b). A *p* value of <0.05 was considered to be significant.

### Fracture prediction

The ability of baseline DBA to predict incident hip fractures was compared versus conventional FN‐BMD, TR‐BMD, and TOT‐BMD to determine whether the use of the full spatial resolution of DXA can help to capture bone strength beyond traditional region‐based BMD values. We compared DBA versus FN‐BMD, TR‐BMD, and TOT‐BMD by classifying subjects into two groups; ie, fractured versus fracture‐free controls. To determine the sensitivity for discrimination of fracture types (FN versus TR), we repeated the experiments for each fracture type separately.

Evaluating classification performance is challenging due to the low proportion of individuals suffering an incident fracture during the follow‐up period, termed *class imbalance*. To address this issue, the precision‐recall characteristics (PRC) plot^(^ ^37^ ^)^ was used besides the receiver operating characteristic (ROC) curve analysis. The area under the curve (AUC) was reported for the ROC and the PRC plots. To determine the 95% confidence interval (CI) and statistical significance of any difference between the fracture versus fracture‐free curve profiles, bootstrapping with *n* = 1000 repetitions was employed.

### Fracture patterns

ROC analysis was employed to classify fractured cases into FN versus TR fractures to determine whether DBA can differentiate between fracture patterns. To further visualize spatially complex fracture‐specific patterns, the component of the BMD texture pattern that is attributable to aging alone, shown in Fig. 1, must be removed. To cancel the aging effect, BMD maps were normalized with respect to their DBA as follows: for each individual BMD map and at each pixel coordinate, the probability of observing a BMD value lower than the given pixel BMD among the population with a similar DBA is reported as a number between 0 and 1. Here, we refer to these normalized BMD maps as quantile maps. Next, the pixel‐level changes in quantile maps were tested using a Mann‐Whitney *U* test between the fracture‐free control group and the fractured cases. To account for the multiple testing issue, computed *p* values were reported as *q* values, defined as the minimum false discovery rate (FDR) level for which a pixel is selected as significant, as described.^(^ ^27^ ^)^ Regions with a *q* value <0.05 were considered significant. All statistical tests were performed in MATLAB v9.7.0.1190202 (R2019b).

## Results

### DBA precision

**Fig. 4:**
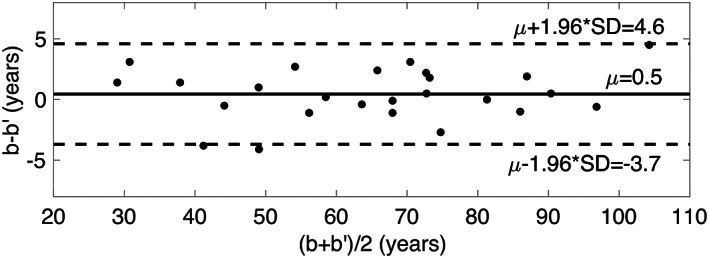
Bland‐Altman plot comparing estimated densitometric bone age before (b) and after ($b^{\prime }$) patient repositioning. Dashed lines represent the 95% confidence interval (mean ± 1.96 SD). The solid black line represents the overall difference (μ). μ = mean bias; SD = standard deviation.

Figure 4 shows the Bland‐Altman plot for estimated DBA for each scan pair (*n* = 25). The coefficient of variation was 2.3%. The mean difference in DBA between the first and second scan measurements was 0.5 years (95% CI, −0.4 to 1.2 years).

### DBA relation with the FN‐BMD *T*‐score and *Z*‐score

**Fig. 5:**
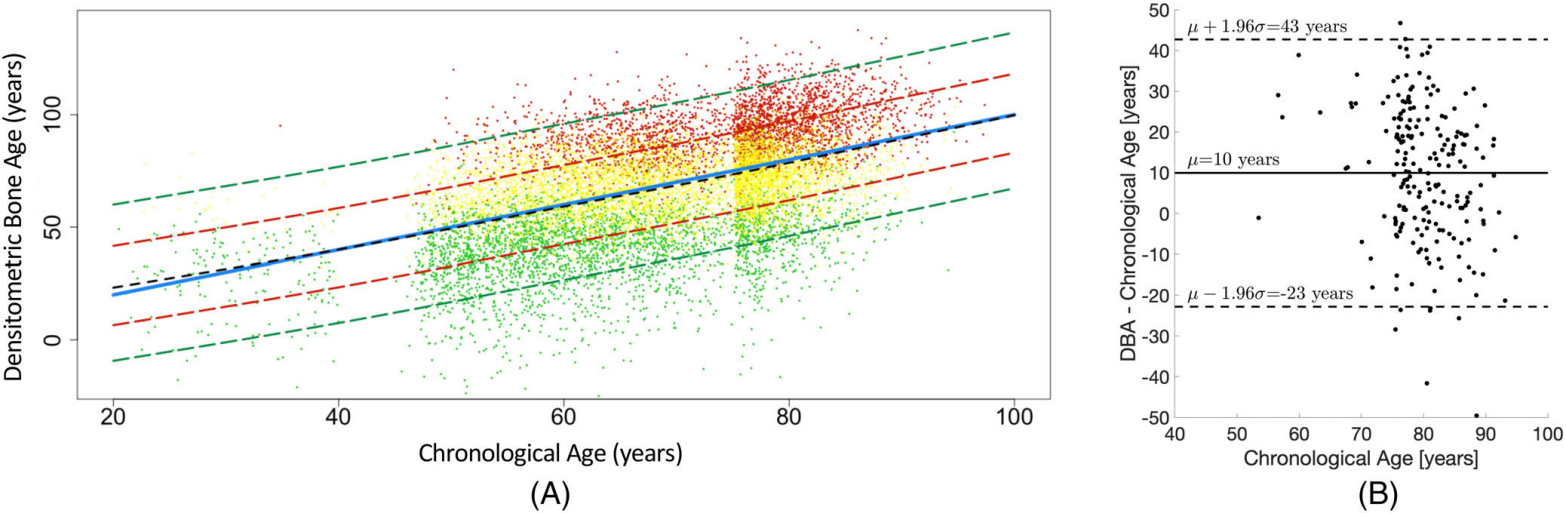
DBA versus chronological age. (*A*) The scattered plot for all cohort subjects. Each green, yellow, or red dot represents one subject from the study cohort categorized by *T*‐score as normal (green), osteopenic (yellow), or osteoporotic (red), respectively. The solid blue line shows the y=x line of equality or identity, and the dashed black line indicates the median DBA as a function of age. The dashed black line almost perfectly follows the blue identity line, demonstrating that DBA equals chronological age on average. Deviation of DBA from the chronological age is proportionate to the *Z*‐score. The red and green dashed lines show *Z*‐scores ±1 and ±2, respectively. (*B*) The distribution of fractured cases in relation to DBA and chronological age. Fractured cases were on average 10 years older in terms of DBA compared to the chronological age. In 72% of fractured cases (150/208), DBA was higher than the chronological age. DBA = densitometric bone age.

Figure 5A,B shows the estimated DBA versus the chronological age across the study cohorts (*n* = 13,338). Each subject is represented with a single dot color‐coded by measured FN‐BMD *T*‐score category: osteoporotic (red; *T*‐score ≤ −2.5), osteopenic (yellow; −2.5 < *T*‐score ≤ −1), or normal (green; *T*‐score > −1). DBA was linearly correlated with both FN‐BMD *T*‐score (*R* ^2^ = −0.82; *p* value < 0.001) and *Z*‐score (*R* ^2^ = 0.78; *p* value < 0.001). *T*‐score was inversely proportional to DBA (as DBA increases along the *y*‐axis, the *T*‐score decreases, also demonstrated by the vertical color variation from green to red among the scattered dots). The *Z*‐score was directly proportionate to the chronological age minus DBA for each subject. Note that at *Z*‐score = 0, the dashed black line follows the solid blue identity line. As points deviate from the identity line, the *Z*‐score increases for points below the identity line and decreases for points above the identity line.

**Fig. 6:**
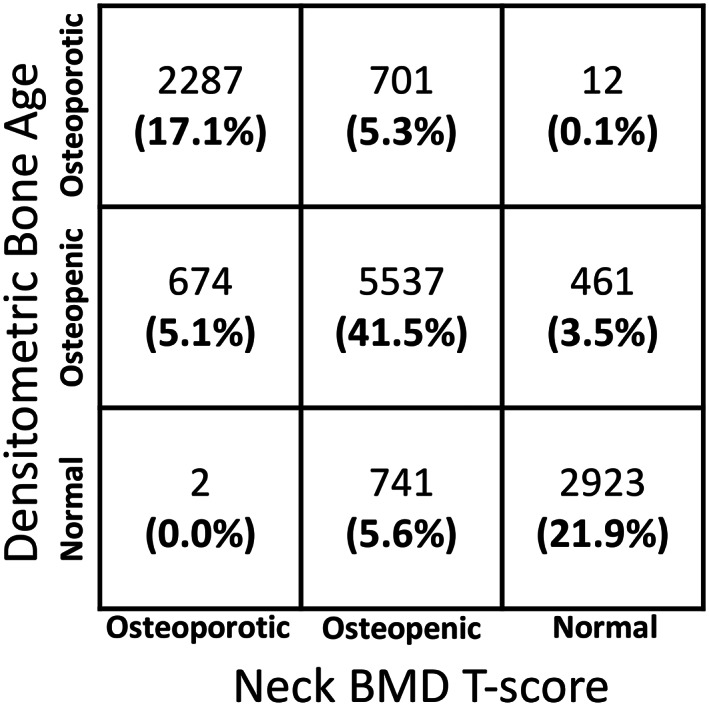
Confusion matrix to assess the consistency between the DBA and the FN aBMD *T*‐score. Using the cutoff thresholds of 83.5 and 54.4 years for DBA, 80.6% of subjects were categorized in the same group as identified by the FN aBMD *T*‐score of −2.5 and −1, respectively. DBA = densitometric bone age.

Given the linear correlation between DBA and FN‐BMD *T*‐score, the corresponding cutoff DBA thresholds for *T*‐scores −2.5 and −1 were 83.5 and 54.4 years, respectively. Figure 6 shows the confusion matrix for classifying subjects into osteoporotic, osteopenic, and normal using DBA versus the FN‐BMD *T*‐score. Few osteoporotic cases were misclassified as normal. This is consistent with observing a clear clustering demarcation line between normal (green dots) and osteoporotic (red dots) subjects in Fig. 5A. Most misclassifications were attributed to the osteopenic cases defined by either DBA or FN‐BMD *T*‐score (Fig. 6). This is observed as yellow dots overlaid on the green and red dots in Fig. 5A. These observations suggest that spatial BMD patterns may provide discriminatory information in cases with intermediate FN‐BMD.

### Fracture prediction

**Fig. 7:**
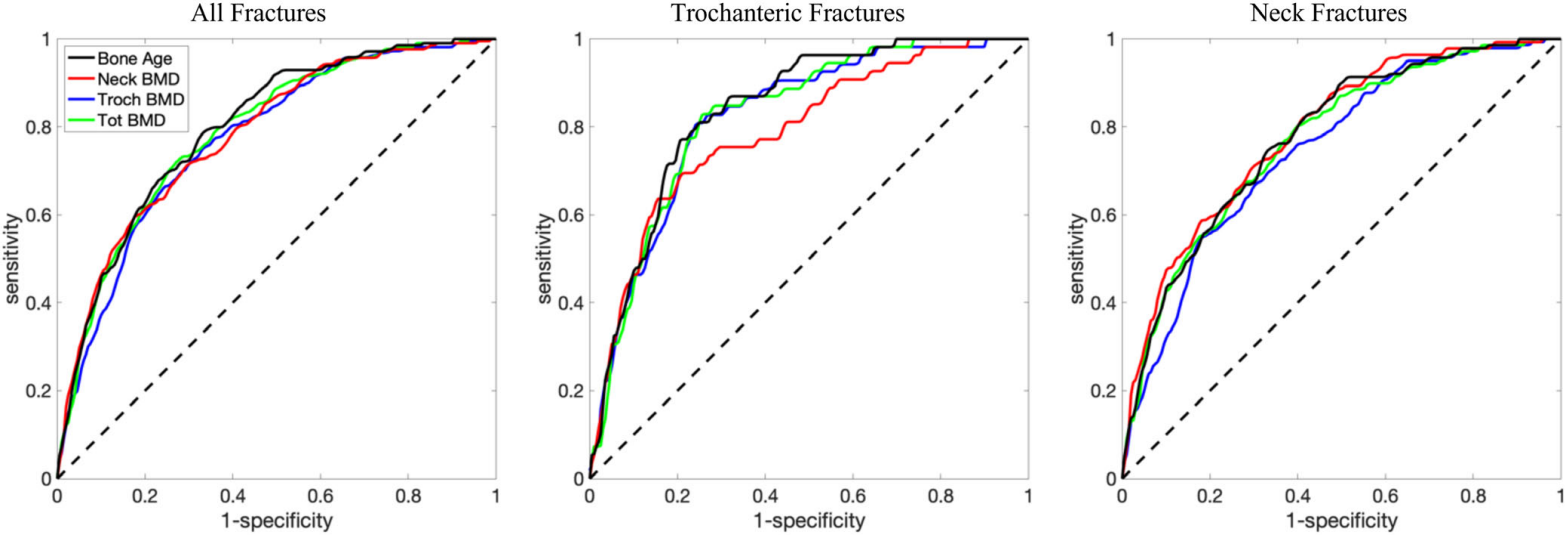
The ROC plots for prediction of fragility fractures. The black dashed line shows the performance of a random classifier, with the solid lines representing densitometric bone age (black), FN‐BMD (red), TR‐BMD (blue), and TOT‐BMD (green). Dotted line indicates random classifier AUC. See Table 2 for the reported AUC values for each graph. A higher AUC indicates better performance. AUC = area under the curve; ROC = receiver operating characteristic.

**Fig. 8:**
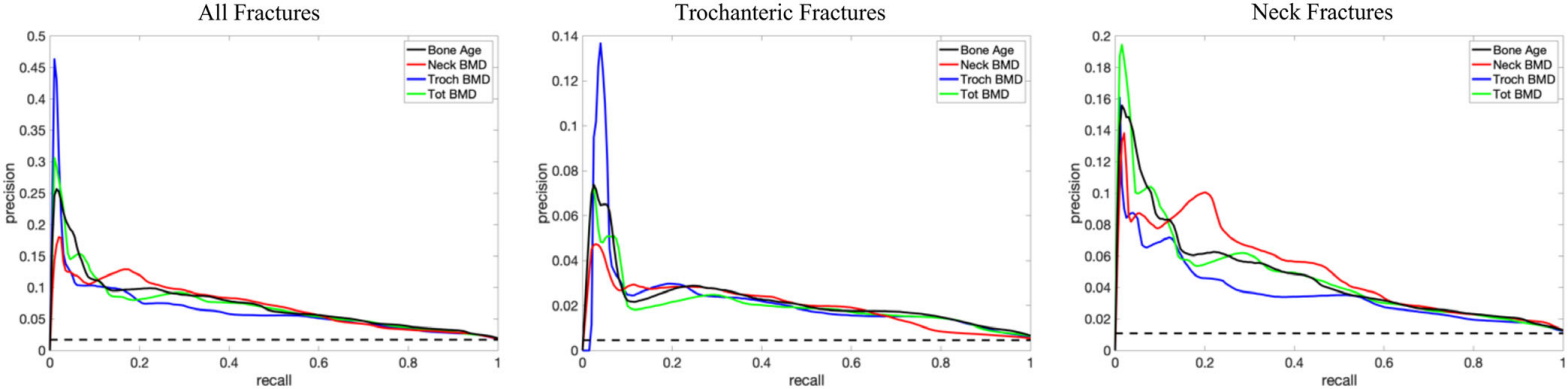
The PRC plots for prediction of fragility fractures. The horizontal dashed black line shows the performance for a random classifier where its height equals the proportion of fractured cases in the population, with the solid lines representing densitometric bone age (black), FN‐BMD (red), TR‐BMD (blue), and TOT‐BMD (green). Dotted line indicates random classifier AUC. See Table 3 for the reported AUC values for each graph. A higher AUC indicates better performance. AUC = area under the curve; PRC = precision‐recall‐characteristics.

Figure 2 provides a visual example of BMD maps for subjects who sustained a follow‐up incident TR fracture (Fig. 2*A*) and a control subject with similar neck BMD and age but remained fracture‐free (Fig. 2*B*). The fractured subject had widespread bone loss in the TR region. This texture variation is reflected in the 18‐year difference in DBA between the fracture versus control subject, despite the same chronological age and FN‐BMD. Tables 2 and 3 show the AUC for the corresponding ROC and PRC plots for quantitative analysis, respectively. In the ROC analysis, AUC = 1 for an ideal classifier and AUC = 0.5 for a random classifier (Fig. 7). In the PRC analysis, AUC = 1 for a perfect classifier and AUC  for a random classifier = the proportion of fractured cases among the cohort (Fig. 8).

**Table 2:** Area Under the Curve for Receiver Operating Characteristic Curve Analysis for the Prediction of Incident Fractures

All cohort	Controls *n*	Number of fractures	DBA	FN‐BMD	TR‐BMD	TOT‐BMD
All fractures	11,691	208	0.799; 95% CI, 0.768–0.824	0.784; 95% CI, 0.751–0.815	0.774; 95% CI, 0.742–0.802	0.789; 95% CI, 0.756–0.816
TR fractures	11,691	52	0.839; 95% CI, 0.795–0.880	0.789; 95% CI, 0.722–0.844	0.823; 95% CI, 0.771–0.869	0.826; 95% CI, 0.775–0.872
FN fractures	11,691	138	0.775; 95% CI, 0.739–0.811	0.786; 95% CI, 0.747–0.821	0.746; 95% CI, 0.704–0.785	0.768; 95% CI, 0.731–0.805

CI = confidence i0nterval; DBA = densitometric bone age; FN = femoral neck; TOT = total hip; TR = trochanteric.

**Table 3:** Area Under the Curve for Precision‐Recall‐Characteristics Analysis for the Prediction of Incident Fractures

All cohort	Controls *n*	Number of fractures	DBA	FN‐BMD	TR‐BMD	TOT‐BMD
All fractures	11,691	208	0.072; 95% CI, 0.053–0.097	0.070; 95% CI, 0.053–0.093	0.063; 95% CI, 0.046–0.085	0.069; 95% CI, 0.051–0.092
TR fractures	11,691	52	0.021; 95% CI, 0.012–0.037	0.019; 95% CI, 0.010–0.031	0.020; 95% CI, 0.011–0.039	0.019; 95% CI, 0.011–0.033
FN fractures	11,691	138	0.046; 95% CI, 0.031–0.066	0.049; 95% CI, 0.035–0.068	0.036; 95% CI, 0.025–0.052	0.046; 95% CI, 0.031–0.066

CI = confidence interval; DBA = densitometric bone age; FN = femoral neck; TOT = total hip; TR = trochanteric.

The ROC analysis for the prediction of both TR and FN fractures suggests a slight increase of approximately 1% (95% CI, 0.4% to 1.6%), 2% (95% CI, −0.5% to 3.5%), and 3% (95% CI, 1.0% to 4.0%) in AUC for DBA in comparison to TOT‐BMD, FN‐BMD, and TR‐BMD, respectively. Compared to FN‐BMD, DBA improved the ROC‐AUC for predicting TR fractures by ~5% (95% CI, 1.1% to 9.8%) with similar performance for detecting FN fractures. Compared to TR‐BMD, DBA improved the ROC‐AUC for predicting FN fractures by ~3% (95% CI, 1.1% to 4.9%) with similar performance to detect TR fractures (Table 2).

The PRC analysis suggested that DBA was more precise than FN‐BMD, TR‐BMD, and TOT‐BMD in the prediction of incident fractures, but it was not statistically significant (Table 3).

### Fracture patterns

**Fig. 9:**
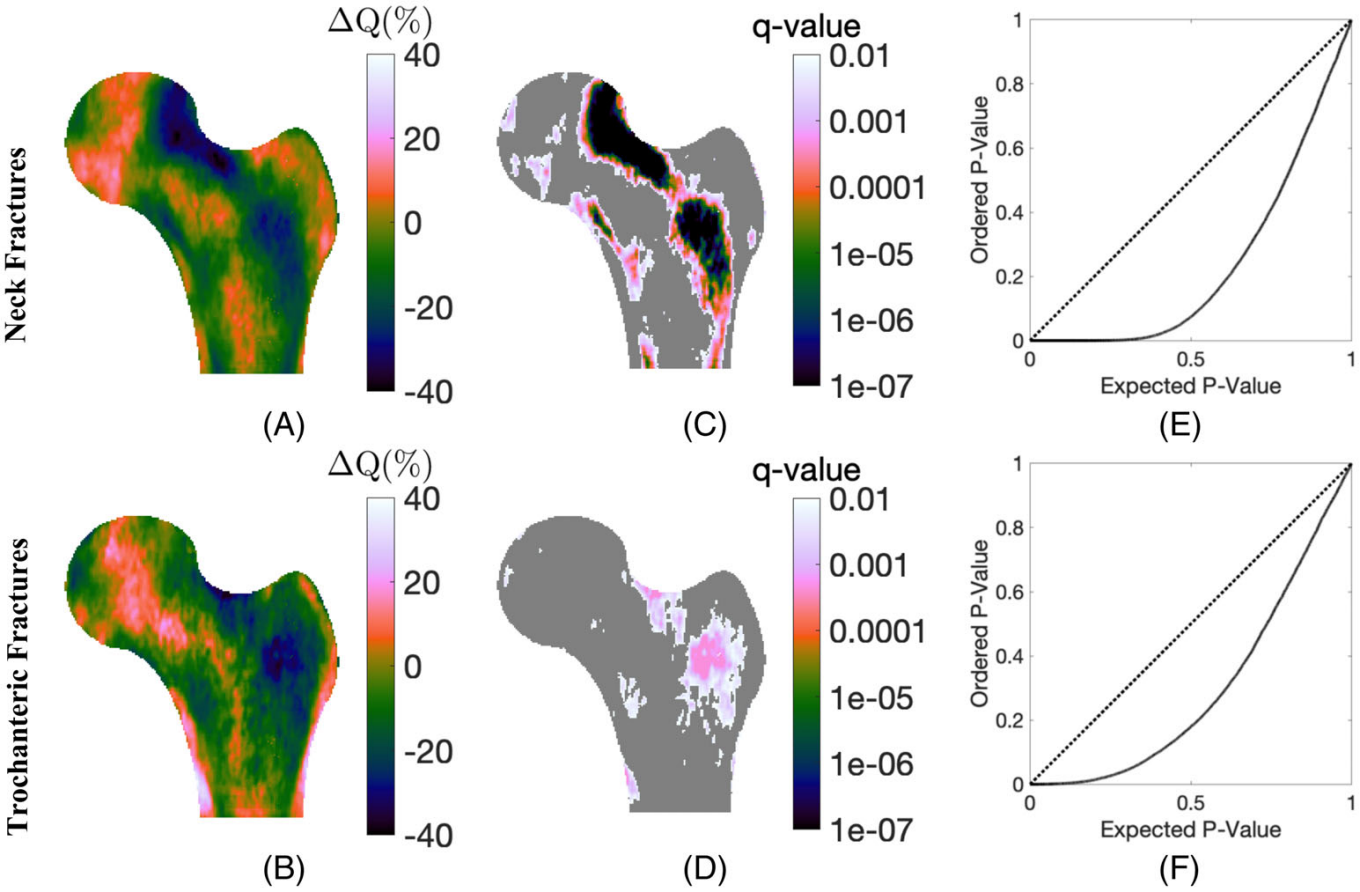
Localizing fracture‐specific patterns using bone‐age normalized BMD maps. (*A,B*) The difference in mean quantile maps between the fracture‐free control groups (*n* = 11,691) and FN fractured cases (*n* = 138) and trochanteric fractured cases (*n* = 52), respectively. (*C,D*) The corresponding statistical significance map using a Mann‐Whitney *U* test followed by FDR analysis. In *C*, a local pattern of BMD deficiency was observed in the same orientation as the principal tensile trabeculae described in plain radiographs of the hip.^(^ ^38^ ^)^ In *D*, widespread BMD deficiency was observed in the trochanteric region. (*E,F*) The PP plot for the FDR analysis. In case of no significant difference, the solid black curve should follow the identity dashed line. FDR = false discovery rate; PP = probability‐probability.

Bone age, unlike FN‐BMD *T*‐score, was not dependent on the potential site of fracture (Tables 1 and 2). Figure 9A‐D show heat maps for the pixel‐by‐pixel difference between controls and fractured groups. The observed fracture‐specific pixel BMD patterns were spatially complex. For neck fractures (Figure 9A,C,E), the *q* map shows a local pattern of bone deficiency that was most apparent in the same orientation as the principal tensile trabeculae first characterized in plain radiograph imaging by Singh and colleagues.^(^ ^38^ ^)^ For TR fractures (Figure 9B,D,F), widespread bone loss in the TR region was observed.

## Discussion

We have examined DBA as a potential new marker of bone quality by quantitating age‐related spatial texture variation in BMD maps using a recently developed spatiotemporal atlas of BMD in the proximal femur.^(^ ^30^ ^)^ We compared the ability of DBA to conventional region‐based BMD measurements including FN‐BMD, TR‐BMD, and TOT‐BMD to predict hip fractures in a large cohort of *n* = 11,899 white women from the MRC‐Hip^(^ ^32^ ^)^ and the UK Biobank studies.^(^ ^39^ ^)^ DBA showed the highest ROC‐AUC, modestly improving the overall performance in comparison to TOT‐BMD by 1% (95% CI, 0.4% to 1.6%), TR‐BMD by 3% (95% CI, 1.0% to 4.0%), and FN‐BMD by 2% (95% CI, −0.5% to 3.5%).

Our findings confirm that region‐specific BMD measurements are most sensitive to fractures occurring within the same anatomical site with relatively worse performance at other locations. FN‐BMD was better than TR‐BMD in identifying FN fractures whereas TR‐BMD performed better for identifying TR fractures, as measured as ROC‐AUC (Table 2). DBA, however, was sensitive to both fracture types, capturing texture patterns in the FN and TR regions simultaneously. Compared to FN‐BMD, DBA improved the ROC‐AUC for predicting TR fractures by ~5%, but with a similar performance for predicting FN fractures. Compared to TR‐BMD, DBA improved the ROC‐AUC for predicting FN fractures by ~3% with similar performance for the prediction of TR fractures. Compared to TOT‐BMD, DBA improved the ROC‐AUC for the prediction of FN and TR fractures by ~0.6% and ~1.4%, respectively.

Our results demonstrated that DBA is a precise quantitative tool, based, as tested by independent analysis of repeat scan acquisitions collected on the same day with patient repositioning between scans. Given its intuitive definition, DBA concept may facilitate patient communication and engagement in clinical practice. This concept is analogous to vascular age^(^ ^40^ ^)^ or brain age^(^ ^41^ ^)^ that have been proposed to express the risk of cardiovascular diseases or dementia, respectively. Note that the terminology “bone age” is not new; it is used by pediatricians to quantitate skeletal maturity in a child and is based on a comparison of a wrist radiograph with atlas patterns to assess the closure of the growth plates.^(^ ^42^, ^43^ ^)^ To avoid confusion, the proposed concept is called densitometric bone age (DBA).

The overall prediction performance of a tool in detecting an event is a function of the prevalence of such events in the population. The population‐attributable risk (PAR) for an incident hip fracture in this study was 52% (versus 28% in the SOF study^(^ ^10^ ^)^) for cohorts with a cutoff *T*‐score of −2.5 and 85% (versus 51% in the SOF study^(^ ^10^ ^)^) for a more conservative cutoff point of −1.5. Unlike the SOF study,^(^ ^10^ ^)^ our findings suggest that a high proportion of proximal femoral fragility fractures (85%) may be attributed to low FN‐BMD *T*‐score. However, note that PAR depends not only on the excess risk imposed by low FN‐BMD but also on the proportion of exposed subjects with a *T*‐score below the cutoff threshold. In our cohort study, the prevalence of osteoporosis was 23.6% (versus 17.7% in the SOF study^(^ ^10^ ^)^) and 61.8% (versus 48.1% in the SOF study^(^ ^10^ ^)^) using the cutoff *T*‐score of −2.5 and −1.5, respectively. The elevated PAR in our study may be attributed to the higher prevalence of subjects with a low FN‐BMD *T*‐score in our cohorts.

Here, we developed a reference aging atlas of BMD textural variation in the whole population and interpreted osteoporosis as a natural process of senescence. DBA was proposed as a surrogate for bone quality in the proximal femur by mapping individual BMD scans to the median aging trajectory. Note that an alternative discriminative learning approach could also be adopted by generating two different aging trajectories for the fractured cases and the fracture‐free controls. We did not adopt this discriminative approach because only a small proportion of the population (~2%) experienced incident hip fractures. Moreover, whether a control subject would remain fracture‐free cannot be guaranteed.

DBA does not per se predict specific fracture‐specific patterns. However, analyzing normalized BMD maps by their corresponding DBA suggests the potential for further improving fracture prediction by incorporating the observed spatially complex fracture patterns. For example, in Fig. 9 those individuals who went on to have an incident FN fracture had a baseline DBA texture pattern of BMD deficiency that was greatest in the distribution of the principal tensile trabeculae whereas those sustaining an incident TR fracture had a baseline DBA texture pattern of BMD deficiency that was evident more diffusely in the TR region. Distinct variation between observed fracture patterns attributed to the FN versus TR fractures thus support the idea that identifying relevant texture features from BMD maps might facilitate fracture risk assessment.

This study also has limitations. First, the follow‐up period for the cohort from the UKBB studies was relatively short, at 3 to 6 years.^(^ ^39^ ^)^ Because fracture information was extracted from Hospital Episodes Statistics (HES) data that was truncated by May 31, 2020, and variable baseline scan time, a variable follow‐up period was inevitable to include the maximum number of participants. Second, although the atlas development methodology is generic and can be readily applied to other ethnicities, our current findings are only applicable to white European women.

In conclusion, the results of this study suggest the potential for improving fracture prediction by analyzing spatial BMD texture patterns. We have shown that the proposed bone age concept is consistent with current diagnostic guidelines but provides a more intuitive reflection than both *T*‐score and *Z*‐score. Besides potential clinical value in facilitating patient communication, we showed that DBA is more precise than FN‐BMD in identifying TR fractures and may facilitate early‐stage fracture risk screening.

## Author contributions

**Mohsen Farzi:** Conceptualization; investigation; methodology; software; validation; visualization. **Jose M. Pozo:** Conceptualization; methodology. **Eugene McCloskey:** Methodology; resources. **Richard Eastell:** Methodology; resources. **Nicholas C. Harvey:** Methodology. **Alejandro F. Frangi:** Conceptualization; methodology; supervision. **Jeremy Mark Wilkinson:** Conceptualization; methodology; supervision.

## Conflicts of Interest

All authors have no conflicts of interest to declare.

### Peer review

The peer review history for this article is available at https://publons.com/publon/10.1002/jbmr.4638.

## Data Availability

The data that support the findings of this study are available from the corresponding author upon reasonable request.
